# *WHO’s Global Age-Friendly Cities Guide*: Its Implications of a Discussion on Social Exclusion among Older Adults

**DOI:** 10.3390/ijerph18158027

**Published:** 2021-07-29

**Authors:** Soondool Chung, Miri Kim, Erica Y. Auh, Nan Sook Park

**Affiliations:** 1 Department of Social Welfare, Ewha Womans University, 52 Ewhayeodae-gil, Seodaemun-gu, Seoul 03760, Korea; sdchung@ewha.ac.kr (S.C.); eauh@ewha.ac.kr (E.Y.A.); 2 School of Social Work, University of South Florida, 4202 E. Fowler Avenue, MHC 1400, Tampa, FL 33620, USA; nanpark@usf.edu

**Keywords:** social exclusion, social inclusion, older adults, age-friendly environment

## Abstract

This study analyzed the World Health Organization’s (WHO) Global Age-Friendly Cities Guide to observe its role in embodying social inclusion of older adults in attempts to prevent social exclusion. Social exclusion refers to the marginalization of individuals and groups from important economic and social opportunities in the society. Many aging societies are implementing social inclusion of older adults as one of their key policy agendas to create a more sustainable and healthy society, in recognition that age functions as one of the essential factors accelerating social exclusion and declining physical and mental health of those affected. In order to explore the pertinence of the WHO guidelines to social inclusion of older adults, content analysis was conducted on each checklist item in the WHO guideline to identify its relation to the four dimensions of social exclusion, which are social interaction, production, consumption, and political engagement. The results showed comprehensive coverage of each dimension by the guideline, although the relative importance of each dimension was unequal. Additional insights were suggested to promote further social inclusion of older adults in the context of an age-friendly environment.

## 1. Introduction

Social exclusion has been a key theme of the European social policy agenda since its emergence in the 1990s [1], and it has been identified in numerous studies as a major issue for the aging population [2,3]. The term ‘social exclusion’ refers to barriers present within a society that restrict important opportunities relevant to livelihood, such as employment, education, housing, and healthcare. Socially excluded individuals or groups experience a lack of resources, goods, services, rights, and participation opportunities and become separated from mainstream society. This can negatively affect any chance of an individual integrating economically and socially within society [4]. In particular, social exclusion of older adults can contribute to detrimental health conditions, such as higher mortality risk, higher morbidity rate, and depression [5].

To engage cities around the world to become more inclusive of the older population, the World Health Organization (WHO) has developed the concept of age-friendly cities. It is designed for older people to participate in social activities without age barriers, and the key characteristics of an age-friendly city are presented in their 2007 Global Age-Friendly Cities Guide. Since the publication of the guideline, it has been applied as a fundamental tool for assessing and improving the age-friendliness of many cities across nations and preventing social exclusion of older adults. Not only has the guideline initiated WHO Global Network of Age-Friendly Cities and Communities, of which 20% of WHO member states have joined, covering 217 million people as of September 2018, it has also served as a basis for many city planning initiatives and national programs on age-friendly environments, providing a focus on the global age-friendly movement [6]. For example, based on the WHO guideline, the Public Health Agency of Canada developed its own Age-Friendly Communities Evaluation Guide to help communities measure the progress of their age-friendly activities [7]. In New York City, the Age-Friendly New York City Project (AF NYC) was initiated to respond to the needs of older New Yorkers, to develop recommendations, and to implement strategies to strengthen the city’s position as an age-friendly city [8]. Similarly, the Seoul Metropolitan Government enacted the “Basic Senior Welfare Act for an Age-Friendly Seoul City” in 2011, and it announced the “Seoul Comprehensive Plans for Senior Citizens” in 2012 as an action plan to realize an age-friendly city, through the application of the WHO guideline [9].

World-wide application of the WHO guideline demonstrates its potential as a global tool in enhancing social inclusion of older adults, a concept which is at the foundation of an age-friendly environment [10,11]. In this context, Menec stressed that social connectivity is the main theme of age-friendly communities to avoid social exclusion. However, the topic of social exclusion of older adults remains largely underexplored [12]. Furthermore, no study has examined the link between social exclusion and age-friendly cities so far, despite the growing number of municipalities that are joining WHO Global Network of Age-Friendly Cities and Communities in recent years [6], and it has been left unclear how the WHO guideline embodies social inclusion. Therefore, this study analyzed the WHO Global Age-Friendly Cities Guide published in 2007 to examine the scope of elements and the multidimensional nature of social inclusion reflected in the guideline by adopting the social exclusion framework of Burchardt and colleagues [13].

## 2. Literature Review

Social exclusion affects people of all ages; however, it has a stronger impact on older adults. Aging demographics, high economic instability, and increasing inequalities among older adults contribute to increased experiences of social exclusion in the older population than for other generations [14,15]. Particularly, in Korea, it is well known that the ratio of older people in Korea who fall below the poverty line has been ranked in first place among OECD countries for many years due to the immature pension systems and the lack of preparation for retirement plans. In addition, in a society where economic productivity serves as a main evaluation indicator, older adults with declining health are treated as burdens, because they are considered to not be productive and therefore no longer contributable to the society [16]. Recognized as one of the social determinants of health, social exclusion places older adults at higher risks of depression, mortality, and morbidity [5]. Moreover, heightened vulnerability to social exclusion in later life is recognized by the following: (1) individuals who have experienced social exclusion in their earlier years are likely to experience further social exclusion when they age; (2) key life events faced in old age, such as the death of a spouse and retirement, can heighten the chances of social exclusion; and (3) ageism and age discrimination prevailing in society exacerbate the marginalization of older adults [17,18]. Older adults may choose not to be included in mainstream society or be excluded for reasons pertaining to individual attributes; however, they may also be excluded from society because a negative perception towards older adults is prevalent in society. For instance, ageism is highly prevalent in many societies around the world, including Korea. Age norms are created and could lead to hindrance in social inclusion, as outcomes like earlier retirement are provoked [19]. Therefore, while addressing social exclusion from the perspective of the individual is significant, the societal context should also be a critical concern in examining the social exclusion of older adults.

Current studies on the social exclusion of older adults have acknowledged its substantial relation to neighborhood. Social relations tend to form within proximity for older adults as they experience retirement and limited mobility with aging. Consequently, they become more reliant on their immediate environment [15,20], and the associated risks of a disabling environment particularly increase with age. Also known as livable cities, elder-friendly cities, and communities for all ages, age-friendly cities modify both physical and social environments to maximize participation opportunities for older adults [21]. Promoting older adults’ participation in social, civic, and economic life, the characteristics of an age-friendly city are closely related to the concept of social inclusion as a countermeasure to social exclusion. Because social inclusion is promoted by ‘building awareness of the impact of social isolation on seniors among seniors, staff, volunteers, and the general public; [embodying] preventive and wellness approaches that address healthy aging; creating age-friendly environments; [and] working against ageism’ [22] (p. 23), it can be argued that social exclusion is prevented by strengthening people’s ability to participate in and remain connected to society. This could be achieved by removing the physical and social barriers that prevent older adults’ participation, and developing adequate infrastructure and community support regarding housing, transportation, and communications.

To guide attainment of age-friendliness, encompassing social inclusion of older adults, WHO made the Global Age-Friendly Cities Guide in 2007, covering eight domains of (1) outdoor spaces and buildings, (2) transportation, (3) housing, (4) social participation, (5) respect and social inclusion, (6) civic participation and employment, (7) communication and information, and (8) community support and health services. Under these eight domains, 169 checklist items were developed and have been used as guiding indicators for creating age-friendly communities as well as measurement indicators for evaluating them. Therefore, checklist items of the WHO Global Age-Friendly Cities Guide are of importance in determining whether the purpose of age-friendliness encompassing social inclusion of older adults has been achieved.

In an age-friendly society, older people are encouraged to engage in productive activities, such as sharing their knowledge and expertise with younger generations and contributing to their communities through employment or voluntary work. In return, their contribution to society is appreciated, and older people are respected [23]. Families with older adults feel less stress when an age-friendly community provides supports and social and health services to older adults. Programs and policies in this type of city are therefore designed to enable older adults to contribute to their communities. Furthermore, communal facilities are designed to be shared and used by people of different ages and interests, leading to harmonious interactions between different generations. In this type of community, even the media depicts older people positively and without the use of stereotypes. In sum, age-friendly cities promote intergenerational exchange and mutual enrichment, which can successfully prevent older adults’ social exclusion. An important link between age-friendly cities and social inclusion has been discussed in several studies [23,24]. However, no previous study has analyzed whether the WHO Global Age-Friendly Cities Guide addresses social inclusion of older adults from the perspective of social exclusion as implied. Therefore, the aim of this study was to analyze the WHO guideline with regards to various domains of social exclusion, with the objective of contributing to higher social inclusion levels of older adults throughout the world.

## 3. Materials and Methods

### 3.1. Social Exclusion Framework for Analyzing Age-Friendly Cities

To analyze the WHO Global Age-Friendly Cities Guide published in 2007 from the perspective of social inclusion of older adults, this study adopted the social exclusion framework of Burchardt et al. [13]. Defining a socially excluded individual as someone who does not participate in key activities in society, Burchardt et al. attempted to develop an empirical measure of social exclusion by constructing the concept of participation using four dimensions of consumption, production, political engagement, and social interaction. Although the selected indicators in their study focused on those of working age, these four dimensions of participation are still highly applicable for demonstrating the social exclusion of older adults, as supported by other studies. For example, consumption, political, and social participation are the core dimensions of social exclusion for older adults in general, according to Phillipson and Scharf [25], and key determinants of inclusion include access to material resources, social relationships, and cultural and civic activities, as described by Gordon et al. [26]. Moreover, how the disadvantages faced by older adults can lead to fewer opportunities to participate in society and loss of roles in later life can be well reflected with the use of Burchardt et al.’s framework [27]. The four dimensions are discussed in detail as follows.

First, the consumption dimension refers to an individual’s capacity to purchase goods and services. Economic exclusion and exclusion from material resources are the most commonly identified exclusion domains for older adults [28,29,30,31]. Economic austerity can both construct and exacerbate old-age exclusion, and resources act as a key determinant of exclusion for older adults [32]. Second, the production dimension considers different opportunities available for participating in economically and/or socially productive activities. Engaging in civic activities, such as volunteering, serves as an important social inclusion domain and is known to augment the well-being and mental health of older adults through the promotion of active aging [28]. Moreover, continued engagement in productive activities is especially significant for older adults as it enables them to gain new identities and social roles that might have diminished over the course of their life due to retirement and structural changes in the family. The third dimension, political engagement, is related to participating in activities involving local and/or national decision-making processes in community-based organizations. These include being a member of a political party, a tenants’ group, a trade union, or a resident group. Any work activity in which an individual engages can be acknowledged for its value to the community and can be considered as a form of political engagement [28]. Fourth, the social interaction dimension refers to the availability of and access to social and familial support. This dimension focuses on relationships with families, friends, and the community at large, which are frequently mentioned as an important factor in determining exclusion. Exclusion in this dimension can serve as a key component in social exclusion, as it is closely related to feelings of loneliness; furthermore, it can indicate whether individuals can sustain a relationship that can help to overcome exclusion as a result of other dimensions [28].

### 3.2. Design and Analysis

The WHO Global Age-Friendly Cities Guide published in 2007 was examined through the lens of Burchardt et al.’s four dimensions of social exclusion [13]. The analysis was conducted in April 2019 by two researchers. Researcher A is a Professor in Social Welfare with research experience of 24 years, whose research area includes age integration, age-friendly environments, active aging, and psychosocial well-being of older adults. Researcher B is a doctoral student in social welfare with six years of research experience regarding age integration, age-friendly environments, and life satisfaction of older adults.

Researchers went through the process of content analysis to identify textual items from the document quantitatively. First, the researchers ruled out checklist items in the WHO guideline that did not explicitly state their applicability for older adults, and they only retained items that mentioned older adults for analysis. Second, each researcher independently coded checklist items with regards to Burchardt et al.’s four dimensions of social exclusion by correlating each item to each dimension [13]. For the social interaction dimension, the researchers included items that expanded availability of and access to social services and support for older adults as they were considered to promote interaction in the community. Furthermore, considering that many older adults face mobility difficulties due to declining health, items that facilitate independent movement were regarded as increasing chances of social support by enabling easy access to services and activities, and were also coded under the social interaction dimension. Moreover, checklist items that foster dissemination of information were coded under the social interaction dimension as well, because appropriate notification can assist older adults in participating in events that otherwise would have been disregarded, and consequently it can increase the chances of participating in interactive activities. Next, for the production dimension, researchers included checklist items that promoted older adults’ engagement in economically and socially productive activities, such as civic participation, volunteering, and employment. Items referring to the provision of diverse options in later life employment were also coded under the production dimension. Next, for the consumption dimension, checklist items that enable older adults to have expanded chances in consuming goods and services within their neighborhood were included. These included items that attempted to increase the purchasing power of adults by providing goods and services that are affordable. Items that referred to reinforcing income were also coded under the consumption dimension. Lastly, in the political engagement dimension, the researchers included items that supported older adults’ participation in the local and national decision-making process. If an item applied to more than one dimension, it was put under all relevant dimensions. Third, the researchers compared their results to identify any discrepancies in their coding and discussed these to reach a consensus. The researchers agreed to only include items that directly accounted for each dimension, as many indirect explanations and subjective inferences could also be drawn. For example, employment options were treated solely as a matter of production even though being employed could induce increase in the consumption level by providing economic stability.

## 4. Results

Due to the purpose of the study, out of 169 checklist items, 86 items were ruled out for having no reference to older adults. Of the remaining 83 items that were in mention of older adults, 63 items were found to be in correspondence with the social exclusion dimensions of Burchardt et al., and they were used for analysis [13]. The results are shown in Table 1.

The results indicate the social interaction dimension to be the most prevalent of the four dimensions, corresponding to 39 of 63 checklist items (62%), and covering all eight topic areas of the WHO guideline. When items in the social interaction dimension were analyzed, efforts to reach out to older adults were observed, exemplified by items like ‘organizations make efforts to engage isolated seniors through … personal visits or telephone calls.’ Next, provision and dissemination of information was observed, exemplified by items like ‘clear and accessible information is provided about the health and social services for older people.’ In addition, utilization of physical infrastructures and atmospheric factors in favor of unrestricted mobility and independent dwelling was observed, exemplified by items like ‘community transport services … are available to take older people to specific events and places.’ Because many older adults have mobility issues, features that assist independent mobility and easy access to services are essential in expanding the ability to engage in social interactions for older adults [33].

The second most prevalent dimension was the production dimension, corresponding to 20 out of 63 checklist items (32%), and covering two topic areas of respect and social inclusion, and civic participation and employment. The items in this dimension endorsed sustained contribution of older adults in productive activities, covering the areas of economic and social participation. Economic participation deals with expansion of employment options through the provision of training opportunities and policy measures to prevent age-based discrimination, marked by items like ‘policy and legislation [preventing] discrimination on the basis of age,’ and ‘retraining opportunities, such as training in new technologies.’ Social participation addresses opportunities for volunteering and civic participation, such as being part of the education process to share their expertise with younger counterparts and having diverse options for voluntary events. Items such as ‘older people are actively and regularly involved in local school activities with children and teachers’ enable them to get involved in diverse areas of work, even after retirement.

The next prevalent dimension was the consumption dimension, corresponding to eight out of 63 checklist items (13%) and covering four topic areas of transportation, housing, social participation, and civic participation and employment. When items in the consumption dimension were analyzed, they were characterized by expanding consumption ability of older adults through the topics of housing, events and activities, and health and social services by lowering the cost of goods or increasing their financial capability. Items such as ‘events, activities, and local attractions are affordable for older participants, with no hidden or additional costs,’ and ‘older workers’ earnings are not deducted from pensions and other forms of income support to which they are entitled’ were observed.

The least prevalent dimension was the political engagement dimension, corresponding to only five out of 63 checklist items (8%) and covering two topic areas of respect and social inclusion and civic participation and employment. The majority of the items in this dimension made efforts to expand and promote older adults’ participation in the local and national decision-making processes, marked by items such as ‘older people are included as full partners in community decision-making affecting them,’ and ‘support exists to enable older people to participate in meetings and civic events.’

## 5. Discussion

This paper examined whether the WHO Global Age-Friendly Cities Guide published in 2007 is relevant in embodying the prevention of social exclusion, particularly in an effort to promote the social inclusion of older adults with the use of Burchardt et al.’s social exclusion framework [13]. The four dimensions of the framework were well reflected in the WHO guideline, suggesting high chances of reducing the risk of social exclusion of older adults with the adoption of the guideline. Considering that the traditional view of social exclusion mostly focused on mitigating financial disabilities, the current multidimensional approach is meaningful in that it expands the discussion of social exclusion to reflect various domains of life in the context of age-friendly environment. Of them all, the social interaction dimension was found to be the most referenced dimension. This not only shows the value that the WHO guideline holds, but also emphasizes the significance of engaging in a meaningful relationship with other people in examining social exclusion. This finding is also consistent with Menec’s emphasis that a basic benefit of age-friendly communities is creating social connectivity [11]. Furthermore, the relational aspect of social exclusion can again be emphasized by the primary motivation of human beings, which is to form and maintain positive close relationships, driven by the need to belong [34]. On that basis, some scholars have come to define social exclusion as a deficit in belongingness [35].

A few themes could be found in the guideline regarding the promotion of social interaction. To begin with, the notion of aging-in-place, which advocates continuous living in the community in which they have resided in younger days, with an appropriate level of independence as an aged person, was exhibited. Since moving to residential care serves as one of the key reasons why the social network of older adults is narrowed, the implementation of aging-in-place can benefit older adults in having greater opportunities of social interaction by utilizing their already well-established connections [36]. An age-friendly environment fosters facilitation of aging-in-place by incorporating all aspects of the physical, social, and service environment to be maintained as a good fit between individuals and their residential setting. The guideline embraces aging-in-place through both direct and indirect references to the services which will help older adults to age in place, and it contains an idea known as a client-centered approach in a social work context. It provides services tailored to the needs and preferences of the older population. Since the older population will not be able to consume, feel socially integrated, or interact with other members of the community when the available services and goods do not meet their needs, the client-centered approach is central to maintaining the life of older adults sustained in their community.

Along with the notion of aging-in-place, more emphasis should be given to constructing meaningful intergenerational solidarity, which has been repeatedly mentioned as one of the critical components in combating social exclusion. For instance, the European Union Treaty of Lisbon holds intergenerational solidarity to be one of the leading objectives in promoting the well-being of European citizens, and it considers it an imperative notion in establishing a sustainable society at a time when demographic changes show an increasingly aging population [37]. In recognizing the positive effects of intergenerational interaction, more cities are deliberately building kindergartens next to nursing homes and holding joint activities in order to facilitate inclusion of older people in the mainstream society. By placing a group of older adults that can be easily disconnected from society adjacent to kindergarten, natural encounters and interactions with a heterogenous group of people are made, making them part of the community [38]. However, intergenerational interaction is only briefly mentioned in the WHO guideline, presenting the need to establish specific intervention methods for practical use.

Furthermore, the WHO guideline can be further enriched in the social interaction dimension by extending the discussion of provision and dissemination of information to close the information gap of older adults, which is evident in today’s information age. This is an imperative issue to address in preventing the social exclusion of older adults, because it is not only a matter of inequality caused by the (in)ability to use technology, but it also offers new means in communicating with others [39]. Especially, information and communication technologies (ICTs) are projected to initiate new opportunities for social participation and social connectedness [40], and hence, further efforts to reduce the digital divide of older adults should be of consideration when local decision makers are endorsing an age-friendly environment. For instance, expanding unmanned systems, such as kiosk machines in Korea, prevent older adults from maintaining their daily life and accelerate the social exclusion process by hindering their active participation in society. Characteristics of kiosk machines, such as small print, poor voice support, and a complex interface, all hamper older adults from operating the machine. Many older adults mention how they no longer visit their regular restaurants or cafes because of feeling uneasy and insecure of facing the complicated steps, failing orders due to making slow decisions, and recognizing that they make other people wait behind in line [16].

Next, the production dimension covers the areas of volunteering, civic participation, and employment comprehensively. Legislative and programmatic responses to maximize economic or social productivity of older adults are suggested in detail. Engaging in productive activities is not only critical in facilitating social inclusion of older adults, but also important for providing them with a sense of purpose and satisfaction, enhancing their psychological well-being [41]. Moreover, the role of media and education in building a respectful atmosphere towards older adults has been observed. Endeavors to create an atmosphere in which older adults are respected can contribute to less ageism and, consequently, expand opportunities for social inclusion.

The consumption dimension deals with the financial ability to consume goods and services. Considering that the traditional view of social exclusion has mostly focused on material factors such as poverty and income, consumption is undeniably a focal issue that can enable social inclusion of older adults [42]. Furthermore, results indicated that over-priced housing could also contribute to spatial segregation, and the inability to participate in events could diminish access to social interaction for older adults. However, the guideline considers a limited range of opportunities that can result in direct economic assistance, which can be a crucial component in promoting social inclusion of older adults [43]. Therefore, along with the provision of inexpensive housing, activities, and health and social services for older adults, more ways that can expand financial capability of older adults are suggested. This is especially crucial in South Korea, where 41.1% of older adults fall below the poverty threshold [44]. Almost half of the older population struggles for the basics of life, and it becomes almost a luxury to be socially involved in community activities and services with such poor purchasing power. Even though the political engagement dimension is the least represented of the four, the guideline nevertheless contains essential aspects related to this dimension. To fully engage older adults in decision-making processes, efforts are suggested to build senior citizen’s organizations, such as the AARP (American Association of Retired Persons). Not only will older adults be socially included, but they will also be empowered by their actions. In addition, local efforts can be made to encourage older adults to vote by providing transportation and helping with the registration process for those with mobility issues or low economic status.

## 6. Limitations

This study has several limitations. First, the effort to recognize and address issues regarding diversity within the older population has been limited in the guideline. Factors such as gender, socioeconomic status, ethnicity, sexual orientation, and health can function to further marginalize an already vulnerable population [12]. However, these factors have been overlooked, and the heterogeneity of older adults should be considered along with measures to reinforce the vulnerability of certain groups within the older population when decision makers are adapting the WHO guideline for more practical use. For instance, actions to involve older adults of low income or minority ethnicities in local and/or decision-making processes should be considered, because they are less likely to be involved [45]. Second, the social exclusion framework by Burchardt et al. does not consider different levels of social exclusion, such as individual, institutional, and societal, and was thus left out in our discussion [13]. Future studies are suggested to reflect different levels of social exclusion in the context of an age-friendly environment to observe what can be done in different sectors or policy spheres to minimize the social exclusion of older adults.

Despite these limitations, the study’s results are meaningful, as various domains of social inclusion that can be applied in supporting active participation of older adults in society have been suggested, which in turn would result in the promotion of longer and healthier lives of the growing older population. In order for the items in the guideline to be realized in practice more actively, the bodies of enforcement are suggested to arrange the guideline into a mandatory form to follow, instead of providing it as a mere recommendation.

## 7. Conclusions

This study analyzed the WHO Global Age-Friendly Cities Guide published in 2007, which provides a central road map in developing age-friendly cities by municipal governments internationally, in multidimensional terms of social exclusion. The results of the study go beyond the social exclusion framework of Burchardt et al. [13] and highlight additional insights provided by the guideline.

In summary, the social interaction dimension is the most referenced dimension, followed by the production dimension, the consumption dimension, and the political engagement dimension. The following observation reflects the relevance of social interaction in promoting social inclusion of older adults, and simultaneously underscores a basic human need in making connections to other human beings. Moreover, crucial central themes of aging-in-place and the client-centered approach are found in the guideline within the social interaction dimension, which allows formerly established social networks to be maintained and services in respect to the specific needs of older adults to be provided. The production dimension covers the areas of volunteering, civic participation, and employment in facilitating opportunities for productive activities sufficiently, along with the role of media and education in creating a society without age discrimination or prejudices through building a respectful atmosphere towards the older population. The consumption dimension, which covers the traditional role of finance in social exclusion, shows room for improvement, as the guideline limitedly encompasses the areas of housing, events and activities, and health and social services, leaving measures for direct economic assistance behind. Lastly, the political engagement dimension captures essential ideas comprehensively, highlighting the importance of engaging in the local and national decision-making process.

In addition to what was covered in the guideline, the discussion was taken further to present suggestions to augment what was lacking in the guideline, and to foster social inclusion of older adults in practical use. Endorsing measures to establish intergenerational solidarity are recommended in the social interaction dimension, since it is frequently mentioned as one of the essential factors in lowering social exclusion. Moreover, reducing the information gap that older adults experience through the utilization of various ICTs are also proposed in the social interaction dimension in response to the rapidly growing information age. Furthermore, measures that support financially difficult older adults are suggested in the consumption dimension, and building senior citizen’s organizations along with promoting voting accessibility are recommended in the political dimension. The results of the study suggest local decision makers and other stakeholders to consider the multidimensionality of social exclusion when adopting the WHO guideline.

## Figures and Tables

**Table 1:** Analysis of *WHO’s (2007) Guideline for Global Age-Friendly Cities* using the framework of social exclusion by Burchardt et al. [13].

Social Exclusion Dimensions	WHO’s Guideline Topic Area (*Sub-Area*) Checklist Items
Social interaction	Outdoor spaces and buildings
	*(Services)* Services are clustered, located in close proximity to where older people live, and can be easily accessed (e.g., are located on the ground floor of their buildings); There are special customer service arrangements for older people, such as separate queues or service counters for older people
	Transportation
	*(Travel destinations)* Public transport is available for older people to reach key destinations, such as hospitals, health centers, public parks, shopping centers, banks, and seniors’ centers *(Information)* Information is provided to older people on how to use public transport and about the range of transport options available *(Community transport)* Community transport services, including volunteer drivers and shuttle services, are available to take older people to specific events and places *(Parking)* Priority parking bays are provided for older people close to buildings and transport stops; Drop-off and pick-up bays close to buildings and transport stops are provided for handicapped and older people
	Housing
	*(Ageing in place)* Older people are well-informed of the services available to help them age in place *(Community integration)* Housing design facilitates continued integration of older people into the community *(Housing options)* There is a range of appropriate services and appropriate amenities and activities in older people’s housing facilities; Older people’s housing is integrated into the surrounding community
	Social participation
	*(Accessibility of events and activities)* The location is convenient for older people in their neighborhoods with affordable, flexible transportation; Older people have the option of participating with a friend or caregiver; Times of events are convenient for older people during the day; Admission to an event is open (e.g., no membership required), and admission, such as ticket purchasing, is a quick, one-stop process that does not require older people to queue for a long time *(Affordability)* Events, activities, and local attractions are affordable for older participants, with no hidden or additional costs (such as transportation costs); Voluntary organizations are supported by the public and private sectors to keep the costs of activities for older people affordable *(Range of events and activities)* A wide variety of activities is available to appeal to a diverse population of older people, each of whom has many potential interests *(Facilities and settings)* Gatherings, including older people, occur in a variety of community locations, such as recreation centers, schools, libraries, community centers in residential neighborhoods, parks, and gardens *(Promotion and awareness of activities)* Activities and events are well-communicated to older people, including information about the activity, its accessibility, and transportation options *(Addressing isolation)* Organizations make efforts to engage isolated seniors through, for example, personal visits or telephone calls
	Respect and social inclusion
	*(Respectful and inclusive services)* Older people are consulted by public, voluntary, and commercial services on ways to serve them better; Public and commercial services provide services and products adapted to older people’s needs and preferences; Services have helpful and courteous staff trained to respond to older people *(Intergenerational and family interactions)* Older people are specifically included in community activities for families *(Public education)* Older people are actively and regularly involved in local school activities with children and teachers; Older people are provided opportunities to share their knowledge, history, and expertise with other generations *(Community inclusion)* Community action to strengthen neighborhood ties and support include older residents as key informants, advisers, actors, and beneficiaries *(Economic inclusion)* Economically disadvantaged older people enjoy access to public, voluntary, and private services and events
	Civic participation and employment
	*(Civic participation)* Older people are encouraged to participate
	Communication and information
	*(Information offer)* Information is disseminated to reach older people close to their homes and where they conduct their usual activities of daily life *(Oral communication)* Oral communication accessible to older people is preferred, for instance through public meetings, community, centers, clubs, and the broadcast media, and through individuals responsible for spreading the word one-to-one
	Community support and health services
	*(Service accessibility)* Residential care facilities, such as retirement homes and nursing homes, are located close to services and residential areas so that residents remain integrated in the larger community; Clear and accessible information is provided about the health and social services for older people; Administrative and service personnel treat older people with respect and sensitivity *(Offer of services)* Health and social services offered address the needs and concerns of older people; Service professionals have appropriate skills and training to communicate with and effectively serve older people *(Voluntary support)* Volunteers of all ages are encouraged and supported to assist older people in a wide range of health and community settings *(Emergency planning and care)* Emergency planning includes older people, taking into account their needs and capacities in preparing for and responding to emergencies
Production	Respect and social inclusion
	*(Public education)* Older people are actively and regularly involved in local school activities with children and teachers; Older people are provided opportunities to share their knowledge, history, and expertise with other generations *(Economic inclusion)* Economically disadvantaged older people enjoy access to public, voluntary, and private services and events
	Civic participation and employment
	*(Volunteering options)* There is a range of options for older volunteers to participate *(Employment options)* There is a range of opportunities for older people to work; Policy and legislation prevent discrimination on the basis of age; Retirement is a choice, not mandatory; There are flexible opportunities, with options for part-time or seasonal employment for older people; There are employment programs and agencies for older workers; Employee organizations (e.g., trade unions) support flexible options, such as part-time and voluntary work, to enable more participation by older workers; Employers are encouraged to employ and retain older workers *(Training)* Training in post-retirement opportunities is provided for older workers; Retraining opportunities, such as training in new technologies, is available to older workers *(Accessibility)* There is support for organizations (e.g., funding or reduced insurance costs) to recruit, train, and retain older volunteers *(Valued contributions)* Employers and organizations are sensitive to the needs of older workers; The benefits of employing older workers are promoted among employers *(Entrepreneurship)* There is support for older entrepreneurs and opportunities for self-employment (e.g., markets to sell farm produce and crafts, small business training, and micro-financing for older workers); Information designed to support small- and home-based business is in a [format] suitable for older workers *(Pay)* Older workers are fairly remunerated for their work; Older workers’ earnings are not deducted from pensions and other forms of income support to which they are entitled
Consumption	Housing
	*(Affordability)* Affordable housing is available for all older people *(Housing options)* Sufficient and affordable housing dedicated to older people is provided in the local area
	Social participation
	*(Accessibility of events and activities)* The location is convenient to older people in their neighborhoods, with affordable, flexible transportation *(Affordability)* Events, activities, and local attractions are affordable for older participants, with no hidden or additional costs (such as transportation costs); Voluntary organizations are supported by the public and private sectors to keep the costs of activities for older people affordable
	Civic participation and employment
	*(Pay)* Older workers are fairly remunerated for their work; Older workers’ earnings are not deducted from pensions and other forms of income support to which they are entitled
Political engagement	Respect and social inclusion
	*(Community inclusion)* Older people are included as full partners in community decision-making affecting them; Community action to strengthen neighborhood ties and support include older residents as key informants, advisers, actors, and beneficiaries
	Civic participation and employment
	*(Civic participation)* Advisory councils, boards of organizations, etc. include older people; Support exists to enable older people to participate in meetings and civic events, such as reserved seating, support for people with disabilities, aids for the hard of hearing, and transportation; Policies, programs, and plans for older people include contributions from older people; Older people are encouraged to participate

## Data Availability

Publicly available datasets were analyzed in this study. This data can be found here: (https://www.who.int/ageing/publications/Global_age_friendly_cities_Guide_English.pdf, accessed on 1 February 2021).
